# Congenital Pouch Colon

**Published:** 2012-07-01

**Authors:** Vivek Gharpure

**Affiliations:** Department of Pediatric surgery, Children’s Surgical Hospital 13, Pushpanagari, Aurangabad, 431001, India

## QUESTIONS

1. What are the diagnostic features of congenital pouch colon (CPC)?

2. What associated anomalies are seen with congenital pouch colon?

3. What are various surgical options in the emergency management of congenital pouch colon?

4. What specific post-colostomy complications are seen with congenital pouch colon?

5. Describe histological features of congenital pouch colon.

6. How does early management differ in female neonates with congenital pouch colon?

7. What short and long term postoperative care is necessary in children with CPC?

8. What are delayed complications after reconstruction for CPC?

## ANSWERS

1. A male patient with CPC often have a wide colovesical fistula and present with anorectal malformation and meconuria; on plain abdominal film, a single large bowel loop occupying more than 50% of the abdominal cavity is also a diagnostic sign. Girls (persistent cloaca/vestibular fistula/anteriorly placed anus etc.) often present late with intractable constipation or multiple episodes of enterocolitis and persistent abdominal distension with common cloaca or anterior ectopic anus/ rectovestibular fistula. The congenital pouch colon can be identified as replacement of a part or entire colon in the configuration of pouch that lacks taenia coli, haustrations, appendices epiploicae, abnormal blood supply and a wide fistula with genitourinary system in a patient of anorectal malformation [1].

2. CPC is often associated with a number of genitourinary and gastrointestinal anomalies nevertheless other system’s anomalies are also reported. Bilateral/unilateral hydroureteronephrosis, renal hypoplasia, vesicoureteric reflux, sacral agenesis, prune belly syndrome, megalourethra, genitourinary tract duplication, hypoplastic bladder, ectopic ureter/kidneys, pseudo-exstrophy bladder, bowel diverticulum, peritoneal bands, absent appendix, rudimentary appendix, duplex appendix, esophageal atresia, cardiac malformations, bicornuate uterus, double uterus, septate vagina, double vagina, hypospadias, abnormalities of vasculature of colon [1,2].

3. Various options that can be opted while dealing a patient of CPC are ileostomy with and without pouch excision (type I and II), colostomy with and without pouch excision (type III and IV), window colostomy in the pouch, tapering of pouch with proximal diversion, tapering and pull through with and without proximal diversion, and pouch excision and pull through with and without proximal diversion etc [1,2].

4. The complications associated with ileostomy in CPC are excoriation, bleeding; electrolyte imbalance, malnutrition, dehydration, vitamin deficiency. The complications associated with window colostomy are prolapse, stenosis, fecal retention, urinary tract infection, pouch dilatation, pouchitis, enterocolitis, persistent abdominal distension, massive mucosal prolapse, para-colostomy hernia and evisceration etc. Boys with CPC often have a wide fistula with urinary tract. Metabolic acidosis and failure to thrive is also seen secondary to reflux of urine in bowel and absorption [1].

5. In most of patients CPC on histopathology shows thinning of muscle layers, outer as well as inner; disorganized muscle layers with normal ganglionosis; however in few cases hypoganglionosis, aganglionosis, neuronal hypoplasia, or presence of heterotopic mucosa can be seen. Such changes have led to some surgeons advocating complete excision of the pouch in all patients with CPC [1].

6. In female neonates with anorectal malformation and CPC, the initial management is largely a stoma formation and deferring the definitive procedure to infancy or early childhood as they are often associated with genitourinary anomalies and not only CPC but the associated anomalies have to be addressed [3,4].

7. Short term postoperative care for CPC involves taking care of perineal excoriations, keeping the stool soft, and preventing stenosis of neo-anus with regular dilatation program. Long term post-operative care of CPC involves intensive bowel management, bowel training, stool softeners, diet modification. These children also require monitoring of their urinary tract, upper and lower for early detection and management of neurogenic bladder, vesicoureteric reflux and other urinary problems. A small number of patients may fail to develop continence and may require Antegrade Enema Procedures. As these patients may not have an appendix and their colons may be too short, catheterizable passage has to create from small bowel. Multivitamins and other nutritional management have to be added for proper growth of the patients [1,2].

8. Common complications of any anorectal reconstruction such as anal stenosis, mucosal prolapse, encopresis, chronic dilatation of retained pouch, chronic fecal retention, retention with overflow and fecal soiling. Dilatation of pouch and fecal overload may cause secondary changes in ureters, kidneys and bladder. Psychosocial problems in patients due to multiple surgical procedures and hospitalization, and encopresis [1].

## Footnotes

**Source of Support:** Nil

**Conflict of Interest:** None declared

